# HIV mRNA Vaccines—Progress and Future Paths

**DOI:** 10.3390/vaccines9020134

**Published:** 2021-02-07

**Authors:** Zekun Mu, Barton F. Haynes, Derek W. Cain

**Affiliations:** 1 Duke Human Vaccine Institute, Duke University School of Medicine, Durham, NC 27710, USA; zekun.mu@duke.edu (Z.M.); barton.haynes@duke.edu (B.F.H.); 2 Department of Immunology, Duke University School of Medicine, Durham, NC 27710, USA

**Keywords:** HIV, vaccine, messenger RNA

## Abstract

The SARS-CoV-2 pandemic introduced the world to a new type of vaccine based on mRNA encapsulated in lipid nanoparticles (LNPs). Instead of delivering antigenic proteins directly, an mRNA-based vaccine relies on the host’s cells to manufacture protein immunogens which, in turn, are targets for antibody and cytotoxic T cell responses. mRNA-based vaccines have been the subject of research for over three decades as a platform to protect against or treat a variety of cancers, amyloidosis and infectious diseases. In this review, we discuss mRNA-based approaches for the generation of prophylactic and therapeutic vaccines to HIV. We examine the special immunological hurdles for a vaccine to elicit broadly neutralizing antibodies and effective T cell responses to HIV. Lastly, we outline an mRNA-based HIV vaccination strategy based on the immunobiology of broadly neutralizing antibody development.

## 1. Introduction

Since the first clinical observation of AIDS and the subsequent isolation of the causing retrovirus HIV in the early 1980s, the HIV/AIDS epidemic continues to be one of the major global health threats despite four decades of intense research [1]. In 2019, there were 1.7 million new infections and 690,000 AIDS-related deaths globally; in 2020, 38 million people were living with HIV [2]. Current prevention and treatment strategies for HIV/AIDS include the use of antiretroviral drugs for pre-exposure prophylaxis (PrEP) and antiretroviral therapy (ART), which have helped transform AIDS from a life-threatening illness to a manageable chronic disease [3]. However, the medications are expensive, require strict adherence to dosing regimens for efficacy and cause side effects. Moreover, some HIV- infected individuals develop drug resistance, requiring changes in medications. Most importantly, access to treatment remains a substantial barrier, particularly in low- and medium-income nations, as evidenced by persistently high rates of new infections over the last ten years [2]. Thus, a prophylactic vaccine remains a central component of a multi-pronged strategy to end the epidemic. Developing an effective HIV vaccine, however, has proven to be extraordinarily difficult. As the epidemic enters its fifth decade, there have been no approved vaccines for HIV and only one promising clinical trial (the RV144 “Thai” trial) that demonstrated a modest 31% efficacy [4].

An ideal HIV vaccine will evoke both humoral and cell-based immunity. Antibodies that neutralize the virus would provide the first layer of defense, preventing infection of host cells upon virus entry into the body. In the event that some virions elude neutralizing antibodies, cytotoxic CD8^+^ T cells would provide a secondary layer of defense, eliminating the earliest infected cells, preventing establishment of a latent reservoir of HIV-infected cells. Indeed, in the rhesus macaque Simian Immunodeficiency Virus (SIV) infection model, immunization with a modified rhesus cytomegalovirus vector expressing SIV genes induces potent CD8^+^ cytotoxic T cells that recognize SIV infected CD4^+^ T cells in the context of MHC-E and eliminates infected cells completely in 50% of macaques [5]. Thus, CD8^+^ T cells can indeed theoretically be a target of an effective vaccine. However, efforts to design vaccines that elicit neutralizing antibodies have encountered both viral and immunological roadblocks that must be overcome for a broadly reactive HIV vaccine to be efficacious.

Recent reviews have covered the progress in HIV immunogen design [6,7] and mRNA vaccine technology as distinct topics [8,9,10] but we felt a comprehensive review at the intersection of these two fields was warranted. Here, we review the challenges involved in developing a broadly neutralizing antibody (bnAb) HIV vaccine and discuss how mRNA vaccines may be useful in overcoming them. We also discuss pre-clinical and clinical work with various mRNA-based methodologies that have raised enthusiasm for this technology as a vaccine platform for HIV and consider their limitations compared to traditional vaccine approaches. We conclude with a proposed strategy for an mRNA-based HIV vaccine.

## 2. The Challenges of Developing an HIV Vaccine

Current HIV vaccine efforts have primarily focused on inducing antibodies that prevent the virus from infecting host cells. Much of the work in this area has been based on protein subunit vaccines, an approach utilizing recombinant viral protein as a target immunogen. The primary roadblocks to HIV bnAb vaccine development include the role of tolerance mechanisms in limiting bnAb B cell precursor development, peripheral anergy and the requirement that bnAbs have very unusual traits to neutralize HIV [4,11]. Moreover, since HIV mutates rapidly and integrates into host genes [12], high levels of long-lasting antibodies against multiple bnAb Env epitopes must be present at the time of infection to prevent virus escape and provide sterilizing immunity [13]. Thus, the task for induction of protective bnAb-mediated immunity is to induce high titers of long-lasting neutralizing antibodies to multiple Env epitopes [13]. It has been suggested that adding a CD8^+^ T cell inducing immunogen or platform to a bnAb vaccine may lower the titers of bnAbs needed for protection [14].

### 2.1. Inducing Broadly Neutralizing Antibodies to HIV

HIV neutralizing antibodies prevent virions from infecting CD4^+^ cells by directly blocking virion entry and membrane fusion [15]. In contrast, non-neutralizing Envelope (Env) antibodies that bind to virus-infected cells have been postulated to protect, albeit in a less potent manner, through Fc-related effector functions such as antibody-dependent cellular cytotoxicity (ADCC) [13]. Indeed, ADCC was one proposed correlate of decreased transmission risk in the RV144 HIV vaccine efficacy trial [16]. However, a follow up trial to RV144 carried out in Africa (HVTN702 or NCT02968849 that induced potent ADCC activity), did not show any efficacy [17]. Two ongoing HIV efficacy trials, HVTN 705 and 706 (NCT03060629, NCT03964415), are also testing the hypothesis that non-neutralizing antibodies are protective. Until these trials are completed, efforts to induce bnAbs remain a top priority.

BnAbs develop in some HIV infected individuals after years of infection [18]. By binding to epitopes on the HIV Env glycoprotein and interfere with interactions with the host receptor CD4 or by preventing the structural rearrangements necessary for membrane fusion, bnAbs block free virions from entering host cells and integrating into the genome [15]. Importantly, bnAbs have the capacity to neutralize not only the particular viral strain infecting that patient (the “autologous” virus) but also other strains of HIV (“heterologous” viruses)–hence, the “broad” in broadly neutralizing antibody [4,15,19]. Evidence that bnAbs can protect against infection comes from studies in humanized mice and non-human primates (NHPs), in which passive administration of bnAbs prevents infection upon exposure to HIV or Simian-HIV (SIV expressing HIV-derived Env), respectively [20,21,22,23]. The observation that bnAbs arise during natural infection from studies of antibody-virus co-evolution has fueled enthusiasm that a vaccine could likewise elicit durable humoral immunity by mimicking natural virus evolution with sequential immunizations [11,24,25].

The Env glycoprotein is heavily glycosylated with a dense cover of host-derived N-glycans shielding the underlying viral protein epitopes from antibody interactions [26]. The extremely low fidelity of HIV reverse transcriptase results in the rapid generation of viral mutants bearing base substitutions, insertions and deletions, additions and losses of glycosylation sites and recombination resulting in viral diversity that has proved in most cases difficult for the host antibody and T cell responses to control [27,28,29]. Thus, a vaccine must elicit antibodies that can accommodate the Env glycan shield yet also bind a variety of viral strains in order to provide protection. To this end, one of the most important read-outs for vaccine-induced antibody responses is the ability of serum from vaccine recipients to neutralize a broad spectrum of Env-pseudotyped viruses [30,31]. An ideal HIV vaccine would elicit serum antibodies that have (1) high potency–the ability to prevent virus infection at low concentrations and (2) high breadth–the capacity to neutralize a high percentage of difficult-to-neutralize “Tier 2” viruses [32].

Despite HIV diversity, a number of conserved epitopes on Env have been identified that, if mutated, negatively impact viral fitness. These regions are the epitopes for bnAbs on the gp120 and gp41 subunits of the Env glycoprotein and comprise the CD4 binding site, the high mannose patch, the Env trimer apex, the membrane proximal external region and the gp120-gp41 interface [33]. However, most antibodies induced by HIV infection or vaccination do not target bnAb epitopes on well-folded or closed, native Env but rather bind non-neutralizing and variable regions of Env present on poorly folded or open Env. As noted above, analyses of bnAbs that bind the neutralizing epitopes have revealed unusual properties that likely restrict the representation, activation, and/or maturation of B cells expressing them as surface B cell receptors (BCRs). Most bnAbs have long third heavy chain complementarity-determining region (HCDR3) loops, a feature that allows the antibody to contend with the Env glycan shield. Unfortunately, the nature of V(D)J recombination as well as tolerance control of such antibodies is such that B cells bearing long HCDR3s are either rarely generated or survive [34,35], thereby limiting the frequency of potential bnAb-bearing precursors in the naïve B cell pool. Many bnAbs also exhibit auto- or polyreactivity, binding to self-antigens in addition to Env [11,24]. B cells expressing auto/polyreactive receptors (and thus having the potential to cause pathology) are normally purged during development or are rendered anergic through mechanisms collectively referred to as immunological tolerance [36,37]. These limitations ensure that B cells expressing BCRs that bind neutralizing epitopes are rarer than those that bind Env non-neutralizing sites, thus representing a significant obstacle for vaccine development.

A critical observation is that bnAbs only arise in some individuals during chronic HIV [18] or Simian-HIV [25,38] infection. There is evidence that HIV infection creates a permissive environment that allows otherwise disfavored B cell precursors to develop [39]. Understanding the immunological conditions that permit bnAbs to develop in some infected individuals and not others, is a topic of intense scrutiny. *Thus, challenge #1 is that B cells with BCRs that bind HIV neutralizing epitopes are rarely generated and/or are auto-/polyreactive and thus are subject to immune tolerance*.

Another key feature of bnAbs is a high frequency of somatic mutations and/or insertions and deletions in the genes encoding immunoglobulin heavy and lights chains [22,40], indicative of extensive maturation within specialized microenvironments called germinal centers. An important discovery in the search for bnAbs was the isolation of Env-specific B cells from large cohorts of chronically infected individuals [4,13,41,42]. By sequencing the immunoglobulin heavy and light chains of individual Env-specific B cells and by analyzing the neutralization potency and breadth of clonal lineage members over time, while simultaneously tracking the co-evolution of the virus, researchers were able to map the biological arms race that facilitates bnAb maturation during chronic HIV infection [41,42,43,44]. In knowing the sequences of different members of a clonal lineage, researchers could computationally infer a phylogenetic tree for a given bnAb lineage, including a germline precursor (also called the unmutated common ancestor) at the “root,” inferred intermediates at “branch points” and sequences recovered from patient B cells as the “leaves” [11,45,46]. The germline precursor represents the sequence of the unmutated immunoglobulin heavy and light chains generated through V(D)J recombination during B-cell development and intermediates represent the likely variants that occurred as the immunoglobulin genes mutated in germinal centers. In essence, a bnAb phylogenetic tree provides a reverse-engineered roadmap of the mutations that took place to turn an unmutated BCR into a bnAb [47,48]. Support for this computational approach lies in the isolation of putative bnAb precursors from healthy people [49,50,51,52] and activation of B cells in transgenic mice bearing germline precursor receptors [53,54,55,56] using germline-targeting proteins. Computational analyses of mutations in multiple bnAb lineages revealed another reason why bnAbs are rare and thus require time to develop during infection: bnAbs are enriched for mutations that are improbable, based on the DNA sequence-dependent activity of the somatic mutation machinery [57]. For example, we have described a particular mutation (G57R) in the immunoglobulin heavy chain of a bnAb germline precursor that is predicted to be an improbable occurrence but it confers neutralization breadth when acquired [57]. Thus, the development of a naïve B cell bearing a receptor with bnAb potential represents a rare event (*Challenge #1*, above) and the acquisition of the correct mutations needed to confer bnAb activity represents a second rare event. *Challenge #2 is that bnAb generation requires high levels of improbable mutations in germinal centers.*

**Figure 1:**
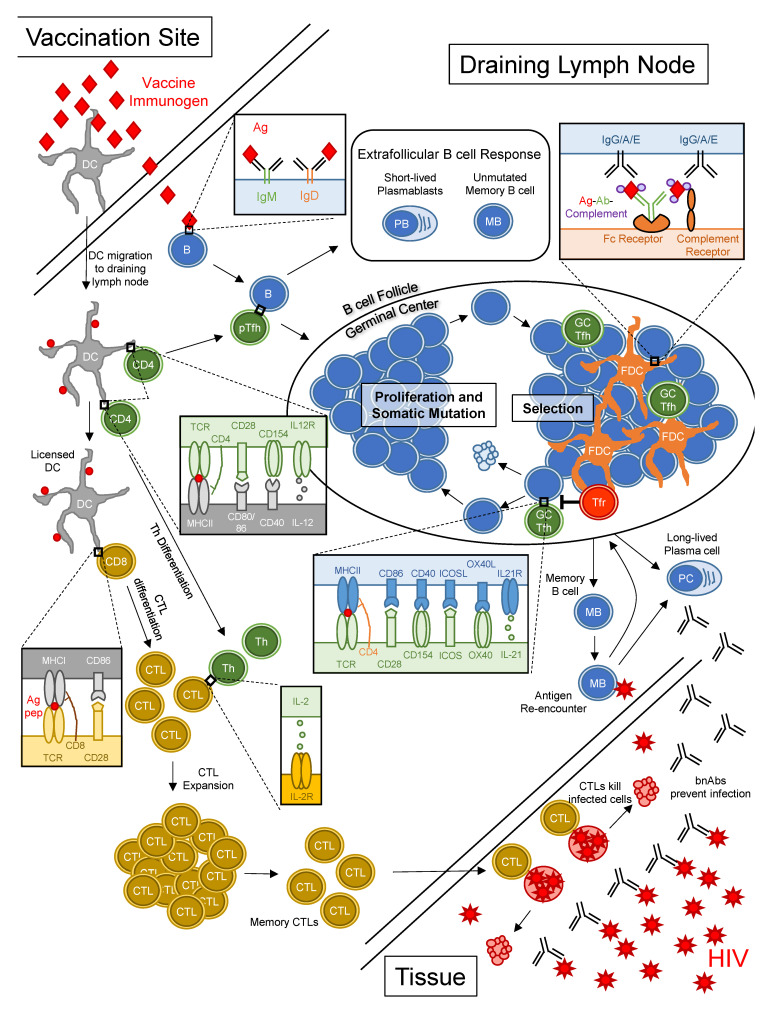
Eliciting humoral and cellular responses through vaccination. Vaccine immunogens prompt antigen-specific responses from B cells and T cells in draining lymph nodes. B cells are blue, CD8^+^ T cells are gold and CD4^+^ T cells are green. Some of the key molecular interactions between different cell types that are needed for optimal humoral and cellular defense are shown in boxes. For optimal humoral responses, antigen-specific B cells must interact with pre-TFH cells (“pTFH”) to initiate germinal center reactions. Within germinal centers, B cells proliferate and undergo somatic mutation, in which mutations are introduced into the genes encoding the B cell receptor, thereby enhancing or diminishing affinity for antigen. B cells bearing receptors that have acquired affinity through mutation interact with TFH cells to receive survival signals and re-enter the cycle of proliferation and mutation. Germinal centers produce long-lived plasma cells, which secrete high affinity antibody and memory B cells, which differentiate into plasma cells upon re-encounter of antigen. For protection from HIV, the goal of vaccination is to induce broadly neutralizing antibodies (“bnAb”), which serve as a first layer of defense by preventing HIV virions from infecting cells. For optimal induction of cellular immune responses, CD8^+^ T cells, dendritic cells and CD4^+^ helper cells are required. CD4^+^ T cells “license” dendritic cells to activate peptide-specific naïve CD8^+^ T cells, which receive co-stimulatory signals from both dendritic cells and CD4^+^ T cells to proliferate and differentiate into Cytotoxic T Lymphocytes (CTLs). For protection from HIV, a second goal of vaccination is to elicit a pool of HIV-specific memory CTLs to serve as a secondary layer of defense, killing host cells that become infected by virions that escape neutralization by bnAbs. Abbreviations: Ag: antigen; CTL: Cytotoxic T Lymphocyte; DC: Dendritic cell; FDC: Follicular Dendritic cell; GC TFH: Germinal Center T follicular Helper cell; pTFH: Pre-T Follicular Helper cell; TFR: T Follicular Regulatory cell.

The biology of bnAb development implies that an effective vaccine will need to induce robust and prolonged germinal center responses to overcome barriers to bnAb maturation. Thus, we will briefly review the biology of germinal centers. Germinal centers are sites in lymphoid tissue where B cells undergo iterative rounds of proliferation, immunoglobulin mutation and selection over a timespan of weeks after vaccination or infection and results in the prolonged production of high affinity antibody and long-lived humoral memory (Figure 1). This process stands in contrast to early B cell responses to antigen in extrafollicular spaces that generate short-lived plasmablasts secreting unmutated antibody [58]. Germinal center responses begin with the binding and activation of a B cell with antigen-specific BCR. In parallel, dendritic cells (DCs) at sites of infection or vaccination capture antigens, migrate to draining lymph nodes and present peptides derived from antigens to CD4^+^ T cells, thereby activating and driving differentiation of peptide-specific pre-follicular helper T (TFH) cells [59]. Peptide-specific pre-TFH cells interact with activated Ag-specific B cells bearing peptides on MHCII, which triggers co-stimulatory interactions between the B cell and pre-TFH cell, setting the stage for germinal center formation [60,61]. The activated B cell starts dividing, thereby forming a nascent germinal center. Within the growing germinal centers, B cells interact with TFH cells, along with antigen-capturing follicular dendritic cells (FDCs) [62,63] and follicular regulatory T (TFR) cells [64,65,66], cycling between phases of somatic hypermutation, in which the immunoglobulin heavy and light chain genes acquire mutations through the enzymatic activity of Activation-induced cytidine deaminase (encoded by gene *AICDA*) [67] and selection based on antigen affinity and peptide presentation to TFH cells [68]. Through repeated cycles of proliferation, mutation and antigen-based selection, the average affinity of BCRs for antigen increases over time, a process referred to as affinity maturation [69,70]. The cellular products of the germinal center response, long-lived plasma cells and memory B cells (Figure 1), exert effector functions and form the basis for enduring humoral immunity.

The presence of heavily mutated immunoglobulin heavy and light chains in bnAbs isolated from HIV+ subjects has been interpreted as a result of prolonged residency (or perhaps multiple residencies) of Env-specific B cells within germinal centers. Thus, successful induction of germinal centers fulfills two goals of HIV vaccination: (1) germinal centers provide the physiologic environment to facilitate the genetic evolution of germline precursors into bnAbs and (2) germinal centers provide the cellular output for durable bnAb production in the form of long-lived plasma cells and memory B cells.

Based on the concept that bnAb and viral evolution are fundamentally coupled processes, a vaccine prime/boost strategy has emerged that is based on sequential injections of optimized immunogens that “shepherd” the vaccine-induced antibody response from germline precursor to bnAb. In essence, the sequential immunization approach represents an attempt to mimic Env evolution that would occur with natural infection by first vaccinating with an immunogen bound by rare germline precursors that recruits them into germinal centers to begin the bnAb maturation process [11,49,52]. Subsequent boosting vaccinations contain modified forms of the priming immunogen that promote the acquisition of key mutations (many of which are improbable) in germinal centers that increase antibody neutralization potency and/or breadth. In contrast to traditional prime/boost strategies, in which the same immunogen is used repeatedly for vaccination, the sequential immunization approach relies on a series of different immunogens with the goal of eventually inducing bnAb(s). Considerable effort has been dedicated to the design of HIV vaccine immunogens with molecular features meant to engage bnAb lineage B cells and we refer the reader to this excellent review [71]. *Challenge #3 is that bnAb induction by vaccination will require multiple immunogens for sequential immunizations.*

### 2.2. Inducing CD8^+^ T Cell Responses to HIV through Vaccination

CD8^+^ CTLs are important for controlling HIV disease, as evidenced by the concurrent rise in HIV-specific CD8^+^ T cells and decline in viremia during acute infection [72,73], the rapid appearance of mutations in immunodominant epitopes recognized by CTLs [74,75,76,77], the association of non-progressive disease in humans with certain human leukocyte antigen class I alleles [78,79] and the rapid rise in viremia in SIV-infected NHPs following CD8^+^ T cell depletion. Moreover, the work of Picker et al. with rhesus cytomegalovirus SIV vaccination demonstrated CD8^+^ T cells can eradicate early SIV infection [80]. Thus, while CTLs do not protect against infection *per se*, the hope is that a vaccine-induced pool of HIV-specific memory CTLs would eliminate the earliest of infected cells, thereby preventing viral replication and widespread dissemination throughout the body (Figure 1) [81].

Vaccine-induced CTL responses depend not only on CD8^+^ T cells but also CD4^+^ T cells and DCs (Figure 1). For optimal CTL induction, antigenic peptides must first be processed and presented on MHCII by DCs to peptide-specific CD4^+^ T cells, which bind via T cell receptors (TCRs) and the CD4 co-receptor. In the subsequent exchange of co-stimulatory signals between the CD4^+^ T cell and DC (Figure 1), the DC becomes “licensed” to activate naïve CD8^+^ T cells [82,83,84,85,86,87]. The CD4^+^ T cell, on the other hand, responds by proliferating and differentiating into a cytokine-producing effector T helper cell. Through a process of cross-presentation, DCs present peptides derived from exogenous antigens on MHCI to CD8^+^ T cells bearing peptide-specific TCRs [88,89,90,91]. Naïve CD8^+^ T cells that recognize peptide-MHCI complexes require two more signals in order to differentiate into cytotoxic effector cells: CD28 on the T cell interacting with CD80 and CD86 on the DC and IL-2 produced by activated T helper cells, which binds the IL-2 receptor on the primed CD8^+^ T cell [92,93,94,95]. With these three signals, a peptide-specific CD8^+^ T cell undergoes rapid clonal expansion and differentiation into effector CTLs, with potent cytotoxic and cytokine-secreting capacity. Following the massive expansion of CTLs, there is a phase of programmed contraction where most effector CTLs undergo apoptosis but leaving behind a small number of memory CTLs [95]. Upon re-encounter of cognate peptide on MHCI, these memory CTLs rapidly proliferate to form a new pool of effector cells to eliminate infected cells (Figure 1) [96].

A CD8^+^ T cell-inducing vaccine faces the same challenge as an antibody-inducing vaccine, in that it must contend with tremendous viral diversity. A number of approaches have been devised to optimize coverage of potential T cell epitopes in vaccine immunogens, many of which rely on computational algorithms [97,98,99,100,101,102]. HIV-derived proteins with greater conservation, including Gag, Pol and Nef, offer the optimal coverage against a variety of viral strains. A successful T cell vaccine will be characterized by (1) inclusion of mosaic proteins comprising peptides from various viral strains; (2) use of concatenated versions of conserved protein regions, thereby maximizing the number of potential epitopes while minimizing variable epitopes; and/or (3) include multiple short peptides with the goal of focusing T cell responses on target epitopes (reviewed in [103]).

Despite the evidence that CD8^+^ T cell activity offers protective effects in animals, the results of T cell vaccine studies in humans have been disappointing. The Merck Step trial (HVTN 502), in which high-risk men were immunized with recombinant adenovirus encoding HIV Gag, Pol and Nef, was halted early due to futility and the possibility that infection rates were higher in the vaccine group [104]. The HVTN 505 trial (NCT00865566), which sought to induce CD8^+^ T cell responses with a DNA prime and adenovirus boost, similarly showed no protection [105]. Nonetheless, the high bar for bnAb-mediated protection and recent discussions of mixtures of T and B cell vaccines have generated new enthusiasm that an effective HIV vaccine could be a combination of T and B cell stimulatory immunogens. *Thus, challenge #4 is that bnAb and* CD8^+^ *T cell responses to HIV are optimally induced* via *different pathways but both may be required for optimal protection from HIV infection*.

## 3. HIV mRNA Vaccine Platforms

### 3.1. Progress in mRNA Technology for HIV Vaccines

An mRNA vaccine is based on the mRNA encoding of an immunogen(s) that is translated into protein upon delivery into host cells. As early as 1989, it was demonstrated that mRNA encapsulated in cationic liposome was able to transfect mouse cells lines [106]. Later, it was found that even direct injection of naked mRNA into mouse skeletal muscle results in protein translation and expression [107,108]. However, using mRNA for therapeutic or vaccine purposes was not feasible because of the instability of RNA molecules, a lack of efficient delivery methods, uncontrollable activation of innate immunity through RNA sensors and difficulties in large-scale manufacturing of mRNA [107,109]. In recent years, technological advances and improved delivery methods have addressed these obstacles and mRNA-based vaccines have emerged as a promising new platform to deliver antigens. Vaccines based on mRNA have several advantages over traditional vaccine platforms, including increased safety, efficacy and ease and speed of manufacture [8,10,110]. In this section, we provide a brief overview of the recent advances in mRNA formulation for vaccines and their exploitation for HIV vaccination.

#### 3.1.1. Non-Amplifying mRNA Vaccines

**Figure 2:**
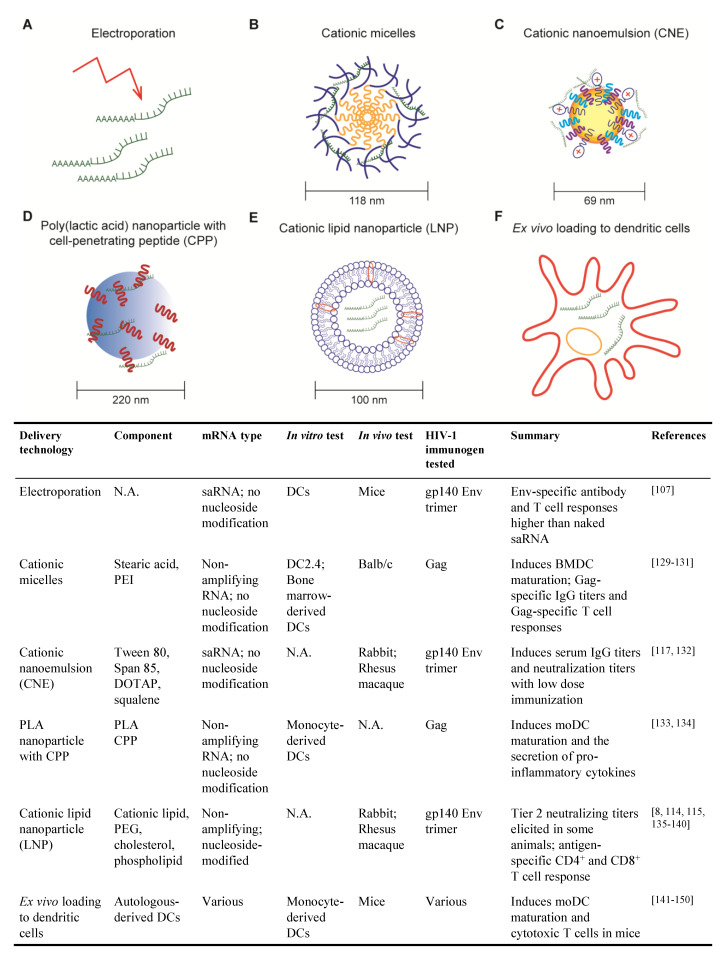
Delivery methods for HIV mRNA vaccine. (**A**) Electroporation; (**B**) Cationic micelles composed of stearic acid (yellow) and Polyethylenimine (PEI) (blue); (**C**) Cationic nanoemulsion (CNE); The yellow core shows squalene. Surfactant such as Tween 80 and Span 85 are shown in blue and purple. Cationic lipid DOTAP (Dioleoyl-3-trimethylammonium propane) is shown with red ‘+’ mark; (**D**) poly(lactic acid) (PLA) nanoparticle (blue core) with cell penetrating peptide (CPP) (shown in red); (**E**) Cationic lipid nanoparticle (LNP); figure shows a lipid bilayer with other components that can be included, such as Polyethylene glycol (PEG), cholesterol or phospholipid; (**F**) Ex vivo loading of dendritic cell (DC). The table below summarizes the HIV mRNA vaccines that have been tested, detailing the delivery method, the type of mRNA, the in vitro and in vivo system used for testing, the mRNA-encoded immunogen and a short note on the results.

Non-amplifying mRNAs, in which the immunogen alone is encoded as an mRNA, represent a simple and economical approach to the development of an mRNA vaccine. Early demonstrations of mRNA-based vaccination relied on non-amplifying mRNAs, injected alone or encapsulated in liposomes, as the source immunogens [111,112]. Substantial progress has been made in recent years to increase both the immunogenicity and safety of vaccines based on non-amplifying mRNA, including optimized codon usage, 3′ capping, 3′ and 5′ untranslated regions, poly-A tail, nucleoside modifications and purification method [8,113]. Non-amplifying mRNAs are amenable to all of the delivery platforms discussed in Section 3.2 and have recently received considerable attention as SARS-CoV-2 vaccines when formulated with nucleoside modifications and encapsulated in lipid nanoparticles [114,115]. Several pre-clinical and clinical studies of HIV mRNA vaccines have been published (Figure 2) and are discussed in more detail in the context of their delivery method in Section 3.2.

#### 3.1.2. Self-Amplifying RNA Vaccines

Self-amplifying RNAs (saRNAs) are replicons engineered from RNA viruses that encode vaccine immunogens as well as viral replication machinery. As such, saRNAs are capable of replicating their RNAs after entering the cell cytosol, thereby enhancing production of the encoded immunogen compared to non-amplifying RNAs (reviewed in [110,116]). Due to the robust production of encoded immunogen, saRNAs exhibit the same level of immunogenicity as non-amplifying RNA at a lower dose [110,116]. Most saRNAs are developed from positive-sense, single-stranded alphaviruses, such as Venezuelan equine encephalitis virus, Sindbis virus and Semliki forest virus. A simple saRNA, encoding RNA-dependent RNA polymerase and an HIV immunogen, for example, can be delivered to cells through approaches detailed in Section 3.2. Alternatively, saRNAs can be delivered as viral replicon particles (VRPs). To generate VRPs, the genetic information for alphavirus structural proteins is replaced with HIV antigen sequences. Supplying the recombinant alphavirus structural proteins in-trans to cell culture results in the packaging of VRPs. Without the genetic information for the structural proteins, VRPs are not capable of generating new infectious viral particles [116]. Several HIV vaccine candidates employing saRNA have been developed and are in testing (Figure 2) [117,118] and are discussed in more detail in Section 3.2.

#### 3.1.3. Nucleoside Modification of mRNA

An ongoing discussion for mRNA vaccination centers on the use of nucleoside modification to minimize recognition of vaccine mRNAs by innate immune sensors [9]. RNA binds to and activates toll-like receptor (TLR) 3, TLR7 and TLR8 on DCs, leading to DC maturation and secretion of anti-viral cytokines, especially type I interferons (IFNs) [119]. Other intracellular virus detection systems, such as the retinoic acid-inducible gene I (RIG-I) and the RIG-I-like receptor (RLR) protein family, RNA-dependent protein kinase (PKR) and the 2-5A system, recognize foreign cytoplasmic RNA and also trigger type I IFN responses, repress RNA translation and promote RNA degradation [120,121]. On the one hand, mRNA-induced anti-viral responses may serve to bridge the innate and adaptive immune responses, thus providing a ”self-adjuvating” effect for mRNA vaccines [122,123]. Conversely, anti-viral IFN responses have been implicated as inhibitors of vaccine-induced antibody and T cell responses [124]. We suspect that the timing and/or magnitude of mRNA-induced innate immune responses has important effects on vaccine-induced antibody and T cell responses and is worth further investigation.

For mRNA vaccines in which excessive innate immune activation is undesired, several chemical approaches have been developed to reduce anti-viral responses to vaccine mRNAs. mRNA with modified nucleosides, such as pseudouridine or methylated nucleosides, exhibits much less activation of TLR3, TLR7 and TLR8 than unmodified mRNA [119]. As a bonus, mRNA containing pseudouridine exhibits enhanced translation compared to unmodified mRNA due to less activation of RNase L and translation suppressor downstream of the 2-5A pathway [121,125,126].

The safety and translational efficiency of vaccine mRNAs can be further optimized by high performance liquid chromatography (HPLC) purification to eliminate double-stranded RNAs, which activate TLR3 [127,128]. Strikingly, HPLC-purified mRNA containing pseudouridine induced even lower levels of pro-inflammatory cytokine secretion compared to unpurified, pseudouridine-modified mRNA in DCs, while the translation level was 10- to 1000-fold higher than unpurified mRNA [128].

### 3.2. Progress in mRNA Delivery Strategies for HIV Vaccines

The lack of efficient delivery technologies for mRNA molecules was a major bottleneck to the development of mRNA-based vaccines. The ideal delivery method will protect mRNA from degradation and facilitate cellular entry with minimal toxicity [8]. mRNA delivery methods that have been tested for HIV vaccine are detailed below and summarized in Figure 2.

#### 3.2.1. Electroporation

Electroporation was among the first techniques developed for the delivery of mRNA vaccines. Cu et al. used mice to show that in situ electroporation delivery of saRNA encoding HIV gp140 Env induced higher Env-specific IgG titers and higher frequencies of Env-specific CD4^+^ and CD8^+^ T cells than naked saRNA [107]. While the direct application of electroporation to patients is not foreseeable, this technique can be exploited to deliver mRNAs into patient-derived DCs for re-infusion (detailed in Section 3.2.6).

#### 3.2.2. Cationic Micelles

Cationic micelles comprising a polyethylenimine-stearic acid copolymer (PSA) were first developed for peptide and protein delivery but were later tested for the delivery of a HIV mRNA vaccine [129,130]. PSA molecules self-assemble into nanoparticles in the aqueous phase, with a hydrophobic core formed by stearic acid and a cationic hydrophilic outer layer formed by polyethylenimine, which enables condensation of anionic mRNA molecules. Cationic micelles efficiently deliver mRNAs to DCs with minimal cell death when used at the proper PSA/mRNA ratio and induce DC maturation ex vivo [130].

To test the efficacy of cationic micelles for HIV mRNA vaccination, mice were immunized with PSA cationic micelles containing non-amplifying mRNA encoding HIV Gag (PSA/mGag) [130]. PSA/mGag immunization induced higher Gag-specific IgG titers than that elicited by naked Gag mRNA and generated Gag-specific IL-4-secreting CD4^+^ T cells and IFN-γ-secreting CD8^+^ T cells.

Cationic micelles have also been tested as a means to deliver mRNAs to mucosal sites, an important consideration for HIV vaccination. Cationic micelles consisting of cyclodextrin-polyethylenimine were employed to deliver HIV gp120 mRNA as an intranasal vaccine to mice [131]. This cationic micelle formulation was able to penetrate epithelial barriers and gp120-specific antibody and T cell responses were evident after two immunizations. Importantly, gp120-specific IgA was detected in both nasal and vaginal washes, indicative of a systemic antibody response that protects distal mucosal sites, an important consideration for sexually transmitted pathogens like HIV.

#### 3.2.3. Cationic Nanoemulsion

Cationic nanoemulsion (CNE) was developed as a delivery system for saRNA vaccines based on the oil-in-water adjuvant MF59 [132]. CNE is prepared by mixing Tween 80 with the surfactant Span 85, the cationic lipid DOTAP and squalene. Squalene forms the core of the particle, surfactant stabilizes the emulsion and cationic lipid enables mRNA binding.

In vitro studies demonstrated that CNE protects mRNA from RNase degradation [132]. saRNA encoding HIV gp140 Env trimer delivered using CNE was compared to three other vaccine platforms in rabbits: saRNA delivery using viral replicon particle (VRP), gp140 Env trimer protein adjuvanted with MF59 and CNE-formulated plasmid DNA [132]. After two immunizations, a low dose of CNE-formulated saRNA elicited higher Env-specific serum IgG titers than those induced by VRP-delivered RNA or MF59-adjuvanted Env protein. Additionally, all animals immunized with CNE-formulated saRNA exhibited higher neutralization titers against tier 1 (easy to neutralize) pseudoviruses.

In rhesus macaques, the CNE-delivered gp140 saRNA vaccine elicited antibodies that neutralize autologous viruses to a greater degree than VRP-based vaccine but less than immunization with gp140 protein adjuvanted with MF59 [117]. Interestingly, macaques primed with the CNE-based vaccine then boosted with the gp140 protein in MF59 mounted equivalent antibody responses to macaques primed and boosted with gp140/MF59. Moreover, the Env-specific T cell response to the CNE-containing vaccine was characterized by IFNγ, IL-2 and IL-4 production, whereas VRP and protein subunit vaccines only induced IL-4.

#### 3.2.4. Poly (lactic acid) Nanoparticle with Cell-Penetrating Peptides

Poly (lactic acid) (PLA) polymer is a biodegradable and biocompatible material that has been extensively studied and approved by the US Food and Drug Administration [133,134]. PLA nanoparticles (PLA-NPs) are able to encapsulate a wide variety of proteins and are efficiently taken up by DCs, thus providing a potential system to deliver mRNAs. However, both PLA-NPs and mRNA molecules are hydrophobic and negatively charged, making it impossible for PLA-NPs to encapsulate mRNAs in the core or carry mRNAs directly on the surface. Thus, PLA-NP with cell penetrating peptides (CPPs) have been developed and tested, where cationic CPPs are used as an intermediate to carry mRNAs on PLA-NPs. In in vitro studies, nanocomplexes of PLA-NP/CPP and HIV Gag mRNA were taken up by monocyte-derived DCs which, in turn, expressed Gag protein, underwent DC maturation and secreted pro-inflammatory cytokines. DC maturation was associated with mRNA-dependent TLR3 and RIG-I signaling [134]. This delivery mechanism is novel and, to our knowledge, in vivo studies using this platform for HIV mRNA vaccination have not been published.

#### 3.2.5. Cationic Lipid Nanoparticle

Cationic lipid nanoparticles (LNP) consist of a lipid bilayer and typically one or more of the following components: polyethylene glycol (PEG), cholesterol and phospholipid [8]. They were first developed to deliver pharmaceutical small interfering RNAs (siRNAs) and entered human clinical trials [135]. LNP exhibit persistent in vivo activity, require lower and less frequent doses compared to other non-viral delivery methods [136]. Excitingly, the safety and immunogenicity of the mRNA-LNP platform in people has been demonstrated by the two SARS-CoV-2 vaccines, mRNA-1273 and BNT162b2 [114,115,137].

Nucleoside-modified, HPLC-purified mRNAs encoding HIV antigens encapsulated in LNP (mRNA-LNP) have been studied in small animal models and NHPs. In mice, an mRNA-LNP vaccine encoding HIV Env induced robust CD8^+^ and CD4^+^ T cell responses [8]. Moreover, in rabbits, vaccination with mRNA-LNP encoding Env gp120 generated anti-gp120 IgG titers six weeks after a single immunization and were further boosted by subsequent immunization [138]. Virus neutralization activity against tier 1 virus and antibody-dependent cellular cytotoxicity (ADCC) were detected in all rabbits after two immunizations. Similar immune responses were observed in rhesus macaques. Strikingly, 50% of the rhesus macaques also generated autologous tier 2 (difficult-to-neutralize) virus neutralization in the serum.

As discussed in Section 2, the induction of HIV bnAbs requires robust and persistent germinal centers which facilitate the maturation of bnAb precursors to full-fledged bnAbs. The ability of mRNA-LNP vaccines to induce such robust humoral response to HIV Env is related to its ability to activate TFH cell response. For example, vaccination of rhesus macaques with mRNA-LNP encoding Env induced robust Env-specific TFH cells, whereas there was little evidence of TFH activity following immunization with a protein Env adjuvanted with the TLR3 agonist poly-ICLC [139]. Thus, the mRNA-LNP platform appears to elicit potent humoral and cellular immune responses to HIV Env.

The mRNA-LNP platform has also been used to deliver bnAbs for passive immunization for HIV [140]. Humanized mice were injected with mRNA-LNP encoding the heavy and light chains of a CD4 binding site bnAb or with the protein antibody. Interestingly, plasma bnAb levels were higher in mRNA-LNP-injected mice than in the mice receiving a large bolus of antibody as protein, indicating that mRNA-LNP offers a strategy to maintain high concentrations of bnAb in vivo. Most importantly, humanized mice administered bnAb-encoding mRNA-LNP were protected from challenge with HIV.

The molecular mechanisms behind the remarkable immunogenicity of mRNA-LNP vaccines are still being untangled but one mystery is the apparent lack of adjuvant. To our knowledge, vaccine studies with mRNA-LNP have all utilized nucleoside-modified, HPLC purified mRNAs, which lack the “self-adjuvanting” properties of unmodified mRNAs and lipid nanoparticles that do not contain any obvious immunostimulants. A clue was provided by Pardi et al., who injected mice with a mixture of influenza HA protein with mRNA-LNP encoding luciferase and observed a robust anti-HA antibody response, suggesting that the LNPs have inherent adjuvant properties [139].

#### 3.2.6. Ex Vivo Loading of Dendritic Cell

So far, we have described mRNA delivery methods in which vaccine recipients are injected with mRNAs (usually in some delivery system), with the hope that some mRNA-encoded immunogens are picked up–or even better, expressed–by DCs. An alternative approach to HIV vaccination involves the removal of monocytes from an individual patient, maturing the monocytes into DCs ex vivo, loading the DCs with mRNA encoding HIV immunogens and then re-infusing the immunogen-loaded DCs back into the patient. This strategy is certainly more labor-intensive and expensive than direct injection of mRNAs but it ensures that DCs are presenting immunogen-derived peptides to activate cognate CD4^+^ and CD8^+^ T cells. Although DCs are able to endocytose naked mRNAs [8], other methods, such as electroporation, are typically used to transfect mRNA more efficiently into DCs. The ability of mRNA-transfected DCs to induce potent antigen-specific CD4^+^ and CD8^+^ T cell responses was demonstrated as early as 2000 with mRNA encoding HIV Gag [141].

To date, most efforts surrounding ex vivo DC loading and HIV vaccination have focused on their potential as a therapeutic vaccine for HIV-infected individuals. Transfection of mRNA encoding HIV genes into DCs confers the ability to activate CD4^+^ and CD8^+^ T cells ex vivo [141,142,143] and multiple clinical trials have been conducted to evaluate ex vivo loaded DCs for their ability to control HIV [144,145,146,147,148]. In most cases, antigen-specific T cell responses were observed, at least transiently but there was little evidence that any of the therapeutic vaccines controlled viremia. While optimistic about the results, researchers in this field continue to optimize the mRNA-DC vaccine strategy to prolong immune responses.

One compelling mRNA vaccine candidate exploits the capacity of mRNA to encode immunogens as well as immunomodulatory factors. “TriMix” comprises mRNAs encoding three DC activating proteins, CD40L, CD70 and a constitutively-active form of TLR4, which is then mixed with mRNAs encoding conserved HIV peptide sequences from Gag, Pol, Vif and Nef [HIVACAT T-cell immunogen (HTI)]. A murine form of the TriMix-HTI mRNA vaccine induced systemic antigen-specific CTLs after direct injection into the lymph nodes of mice and the human version of the mRNA vaccine induced monocyte-derived DCs to mature and induce T cell proliferation and cytokines ex vivo [149]. TriMix-HTI was evaluated as a therapeutic vaccine in ART-treated HIV infected individuals in a Phase IIa clinical trial [150]. Patients were vaccinated with three doses of naked HTI-TriMix, TriMix mRNAs or placebo followed by ART interruption. The vaccine candidate was proved safe and well-tolerated in humans. However, no efficacy in controlling viral rebound was observed. Unfortunately, an extra start codon was mistakenly included in the DNA plasmid template for the mRNA encoding HIV-specific peptides, which may have affected the expression of the immunogen peptides.

Although ex vivo loading of DCs was the first to be evaluated in human clinical trials for HIV mRNA vaccine, this method involves working with autologous DCs, which requires much expertise in cell therapeutics and is laborious and expensive [122]. The research focus has mostly switched to other chemical, synthetic delivery vehicles.

## 4. Overcoming the Challenges to HIV Vaccination with mRNA-Based Approaches

Efforts to develop an effective HIV vaccine over the last forty years have largely served to reveal the roadblocks that a successful vaccine will need to overcome. Vaccines based on mRNA exhibit special properties that may be exploited for a new generation of HIV vaccines. Here, we summarize the immunological and viral challenges to HIV vaccination outlined in Section 2 and list the empirical observations that support the use of mRNA-based approaches to address them.

### 4.1. Challenge #1: B Cells with BCRs That Bind HIV Neutralizing Epitopes Are Rarely Generated and/or Are Auto-/Polyreactive and Thus Are Subject to Immune Tolerance

A key strategy to activating rare, possibly anergic, bnAb precursor B cells is providing well-folded, native-like immunogens in a manner that maximizes their activation and proliferation. Traditional subunit vaccines deliver immunogens as a bolus of protein that, immediately upon injection, is subject to degradation. In contrast, mRNA-based vaccines induce weeks-long production of immunogens in situ [139], increasing the chance that rare bnAb precursor B cells will activate and proliferate after immunization.

Strategies that enhance immunogenicity of HIV Env antigens increase the chance that rare B cells bearing germline precursors with bnAb potential will be activated. One approach to increasing Env immunogenicity is to display Env immunogens on particulate arrays, such as nanoparticles or virus-like particles [151,152]. An antigen multimerization modality that is amenable to mRNA vaccine platform is the encoding of a self-assembling scaffold protein, such as a ferritin nanoparticle, in addition to the Env immunogen [153]. Multimerization of Env on ferritin nanoparticles improves immunogenicity of Env in animal models [54,154,155], thereby enhancing activation of rare and/or anergic bnAb germline precursors.

As discussed earlier, an effective HIV vaccine may need to induce an immunological environment that is permissive for the development and maturation of auto/poly-reactive B cells with bnAb potential, a state akin to that occurring with chronic HIV infection. mRNA-based vaccines have the potential to transiently alter the host immunological milieu through the encoding of immunomodulatory factors. The capacity for an mRNA platform to generate bioactive molecules was demonstrated by Kariko et al., who injected mice with erythropoietin encoded as mRNAs and encapsulated in lipid nanoparticles and observed enhanced erythropoiesis [156]. A variety of immunomodulatory proteins to transiently affect vaccine-induced immune responses could be exploited, including co-stimulatory molecules that enhance DC or lymphocyte activation [149], cytokines that shape T helper responses, antagonistic antibodies that block inhibitory pathways or agonistic antibodies that enhance immunostimulatory signals [157]. While this field is certainly in its infancy, there is already precedence for mRNA-based delivery of vaccine immunogens alongside immunomodulatory molecules that enhance DC activation [149].

### 4.2. Challenge #2: bnAb Generation Requires High Levels of Improbable Mutations in Germinal Centers

A key feature of mRNA vaccines, particularly those delivered via cationic LNPs, is the induction of potent antibody responses via germinal centers. While the molecular basis for the robust induction of germinal centers with mRNA vaccines is still being unraveled, the generation of antigen-specific TFH cells is likely a paramount feature [139,158]. As discussed in Section 3.2.5, lipid nanoparticles exhibit inherent adjuvant properties, which likely contributes to the induction of germinal centers. Additionally, it has been shown that mRNAs are expressed in host cells at high level for up to 10 days after injection [135]. We suspect that prolonged production of mRNA-encoded immunogens in vivo, in contrast to a bolus injection of protein immunogen, works much like antigen release by osmotic pump, in which slow delivery of antigen enhances germinal center activity and induction of HIV neutralizing antibody [159]. Moreover, the in vivo production of immunogen by mRNA vaccination ensures that well-folded, native-like Env proteins and not degraded or biochemically modified forms of Env, are still being produced in situ when germinal centers occur in the weeks after immunization. Indeed, a recent study demonstrated that immunization of rhesus macaques with mRNA-LNPs encoding HIV gp160 Env elicited a durable neutralizing antibody response, lasting at least 42 weeks (the last time point of the study) with no evidence of decay, likely reflecting the generation of long-lived plasma cells in germinal centers [160].

### 4.3. Challenge #3: bnAb Induction by Vaccination will Require Multiple Immunogens for Sequential Immunizations

Sequential immunization with optimized HIV Env immunogens represents a considerable jump in complexity compared to traditional vaccine regimens. On top of the biological complexities of identifying and testing a variety of immunogens is the pragmatic issue of producing them under Good Manufacturing Practices (GMP). In this area, mRNA-based vaccines have clear advantages over protein subunit or live/inactivated virus platforms. The mRNA manufacturing process is quicker and simpler than production systems based on mammalian cells or eggs, has high yield and purity and is scalable [8]. Moreover, all mRNA reaction components are available from commercial vendors as animal component-free reagents, circumventing safety issues that often arise with cell culture-based vaccine components.

### 4.4. Challenge #4: bnAb and CD8^+^ T Cell Responses to HIV Are Optimally Induced via Different Pathways But Both May Be Required for Optimal Protection from HIV Infection

While an effective HIV vaccine will likely need to elicit both bnAb and CD8^+^ T cell responses, vaccine researchers have typically relied on the delivery platform best suited for inducing a humoral vs. cellular immune response. For vaccines based on Env immunogens to elicit antibody responses, the protein subunit approach is most commonly employed: recombinant proteins are injected along with a suitable adjuvant, which favors the induction of TFH cells, germinal centers and antibodies. In contrast, studies of T cell vaccines have relied on delivery of HIV proteins or peptides via DNA plasmids and/or recombinant viruses, in which viral peptides are produced endogenously by host cells and loaded onto MHCI for presentation to CD8^+^ T cells [161,162]. mRNA-based vaccination appears to offer the potential for inducing both humoral and cellular immune responses, although more work is needed to address questions surrounding mRNA format and delivery. For example, the use of unmodified mRNA, which features self-adjuvanting properties, elicits strong CD8^+^ T cell response against tumor antigens, yet studies with these vaccines have rarely investigated the associated humoral response [9]. Likewise, vaccines based on nucleoside-modified mRNA in LNPs evoke potent antibody responses but their capacity to induce CD8^+^ T cell response is less clear [9]. Indeed, one COVID-19 mRNA vaccine (BNT162b2) induces antigen-specific CD8^+^ T cell response in humans [163,164] but subjects vaccinated with a different COVID-19 mRNA vaccine (mRNA-1273) exhibited low to undetectable levels of CD8^+^ T cell activity [114,137]. Thus, more work is needed on the basic immunology of mRNA-based vaccination to decode the properties that dictate induction of humoral and cellular responses.

### 4.5. Unanswered Questions Surrounding mRNA Vaccines

While research innovations have driven rapid progress in the field of mRNA vaccines in the past few years, many unanswered questions and challenges remain in developing an mRNA HIV vaccine [9]. First, the detailed mechanisms of the immunogenicity of mRNA are lacking. For example, what are the cell types that express the mRNA and produce the encoded immunogen? And how do mRNA-encoded immunogens interact with various cells of the immune system? What are the kinetics and magnitude of mRNA-encoded immunogen expression in vivo, especially with complex HIV antigens? Answers to these questions will provide meaningful guidance to the design and evaluation of mRNA HIV vaccines.

An important component of any bnAb-inducing HIV vaccine will be well-folded, native-like Env immunogens in the pre-fusion conformation [4]. For protein subunit vaccines, well-folded Env trimers can be purified prior to immunization. When delivered as mRNA, however, there is no opportunity for purification of well-folded Env trimer protein. To overcome this challenge, well-designed Env immunogens with optimized stabilizing strategies are needed [165,166,167], the feasibility of which was recently demonstrated in mRNA-based SARS-CoV-2 vaccines [137,164,168].

Another important consideration for HIV immunogen design for mRNA vaccination centers on the inescapable dependence on host cells to translate mRNA-encoded immunogens and apply post-translational modifications. For example, a recent study reported on the design of an Env immunogen targeting the purported germline precursor for a bnAb lineage that bound best when produced in a cell line lacking the glycosylation machinery to add complex carbohydrates [56]. This type of immunogen would not be optimal in an mRNA-based vaccine, as host cells would add the undesired carbohydrates in the course of normal protein production. During the early decision-making process for producing a vaccine as an mRNA or protein, it will be important to consider the form of the mRNA-encoded protein product that will be produced by host cells.

Finally, with the recent rare anaphylactoid events to COVID-19 vaccine mRNA-LNP administration that have been attributed to possible sensitivity to polyethylene glycol 2000 in LNPs [169], it remains unclear how frequent such reactions will occur in the future with wide-spread use of mRNA-LNPs.

## 5. Future Directions

Because of the biology of bnAb development, an effective HIV vaccine will likely be more complicated than current vaccines. We envision a series of Env-based immunogens that, when encoded and delivered as mRNAs, will guide B cell maturation from germline precursor to bnAb in germinal centers, through sequential immunization [13]. Due to HIV’s ability to escape from antibody responses, it will be important that a vaccine elicits multiple bnAb lineages that target different conserved sites on the Env glycoprotein and thus may require multiple series of immunogens. For example, one set of immunogens may guide the maturation of bnAbs that bind the CD4 binding site, another set of immunogens may drive a bnAb lineage that binds the Env trimer apex. An important point here is that a multi-bnAb lineage, sequential immunization strategy will potentially require multiple immunogens.

For broad coverage of potential T cell epitopes, the mRNA vaccine could also encode select regions of HIV Gag, Nef or Pol, either as full proteins, short peptides or mosaics. With proven efficacy and safety, immunomodulatory proteins could also be encoded as mRNA to transiently boost immune responses following vaccination. To achieve these goals, vaccine mRNA candidates will have to be designed and tested pre-clinically, produced under GMP and then evaluated iteratively in phase 1 clinical trials. We can imagine an mRNA HIV vaccine regimen targeting two different bnAb lineages with four sequential immunogens per lineage, plus two HIV potential T cell epitopes, encoded as mRNAs. The rapid manufacturing time of GMP mRNA could allow for multiple immunogens to be evaluated in human clinical trials concurrently [8,170,171]. In summary, now that the biology of bnAb development has been elucidated in HIV-infected individuals, the challenges for bnAb development by vaccines are now apparent. The attributes of mRNA vaccines of durability of responses, ease in production, ability to encode complex protein designs and safety as immunogens make them a prime platform for HIV vaccine development.
